# Whole Exome Sequencing Identified a Novel Heterozygous Mutation in *HMBS* Gene in a Chinese Patient With Acute Intermittent Porphyria With Rare Type of Mild Anemia

**DOI:** 10.3389/fgene.2018.00129

**Published:** 2018-04-20

**Authors:** Yongjiang Zheng, Jiehua Xu, Shengran Liang, Dongjun Lin, Santasree Banerjee

**Affiliations:** ^1^Department of Hematology, The Third Affiliated Hospital of Sun Yat-sen University, Guangzhou, China; ^2^Department of Nuclear Medicine, The Third Affiliated Hospital of Sun Yat-sen University, Guangzhou, China; ^3^School of Life Sciences and Biopharmaceuticals, Guangdong Pharmaceutical University, Guangzhou, China; ^4^Department of Cell Biology and Medical Genetics, School of Medicine, Zhejiang University, Hangzhou, China

**Keywords:** acute intermittent porphyria, *HMBS* gene, heme biosynthetic enzyme, novel mutation, heterozygous

## Abstract

Acute intermittent porphyria (AIP) is a rare hereditary metabolic disease with an autosomal dominant mode of inheritance. Germline mutations of *HMBS* gene causes AIP. Mutation of *HMBS* gene results into the partial deficiency of the heme biosynthetic enzyme hydroxymethylbilane synthase. AIP is clinically manifested with abdominal pain, vomiting, and neurological complaints. Additionally, an extreme phenotypic heterogeneity has been reported in AIP patients with mutations in *HMBS* gene. Here, we investigated a Chinese patient with AIP. The proband is a 28-year-old Chinese male manifested with severe stomach ache, constipation, nausea and depression. Proband’s father and mother is normal. Proband’s blood sample was collected and genomic DNA was extracted. Whole exome sequencing and Sanger sequencing identified a heterozygous novel single nucleotide deletion (c.809delC) in exon 12 of *HMBS* gene in the proband. This mutation leads to frameshift followed by formation of a truncated (p.Ala270Valfs^∗^2) HMBS protein with 272 amino acids comparing with the wild type HMBS protein of 361 amino acids. This mutation has not been found in proband’s unaffected parents as well as in 100 healthy normal control. According to the variant interpretation guidelines of American College of Medical Genetics and Genomics (ACMG), this variant is classified as *“likely pathogenic”* variant. Our findings expand the mutational spectra of *HMBS* gene related AIP which are significant for screening and genetic diagnosis for AIP.

## Introduction

Acute intermittent porphyria (AIP) [MIM# 176000] is a rare hereditary metabolic disorder with an autosomal dominant mode of inheritance. AIP is caused by the germline mutation of *HMBS* gene encoding the heme biosynthetic enzyme hydroxymethylbilane synthase which is the is the third enzyme in the heme biosynthetic pathway (Puy et al., 2010). In mitochondria, heme is produced by eight evolutionarily highly conserved enzymes by a complex cellular machinery (Hrdinka et al., 2006). Patients with AIP are characterized by the partial deficiency of the hydroxymethylbilane synthase. However, due to extreme phenotypic heterogeneity, the clinical diagnosis and proper treatment of AIP in China is highly challenging (Yang et al., 2015). The clinical diagnosis of AIP has been made according to the clinical symptoms of the patient and biochemical examination. The level of porphobilinogen (PBG) in urine is a biochemical marker for the clinical diagnosis of AIP. Recently, whole exome sequencing is reported to be the best way to unveil the molecular genetic screening of the AIP patients (Hrdinka et al., 2006). Whole exome sequencing is essential for clinical diagnosis for AIP (Yang et al., 2015). In addition, over 360 *HMBS* gene mutations have been reported to be associated with AIP^1^, rarely been reported in the Chinese population (Anderson et al., 2005; Whatley and Badminton, 2013). Clinically AIP is usually manifested with severe abdominal or stomach ache, accompanied by nausea, constipation, and abdominal distention. Patients with AIP are also presented with several behavioral changes such as irritability, insomnia, and depression (Hrdinka et al., 2006).

In our present study, we identified a Chinese patient with AIP. After clinical diagnosis based on clinical symptoms and biochemical test results, we undertaken a whole exome sequencing in order to understand the molecular genetic basis of the disease in this present case. Whole exome sequencing and Sanger sequencing identified, a single nucleotide deletion (c.809delC) in exon 12 of *HMBS* gene followed by the formation of a truncated (p.Ala270Valfs^∗^2) HMBS protein in this proband.

## Case Report

The proband was a Chinese man of 28 years of age belongs to a non-consanguineous Chinese parent. Two years ago, the proband was visited our hospital with abdominal ache, accompanied by nausea, constipation. Proband has high blood pressure (165/105 mmHg) with a heart rate of 101 beats/min. Proband’s serum sodium concentration was lower than normal (127 mmol/L), with no other abnormal findings. The result of abdomen X-ray and gastro-endoscopy are also normal. After half a year, he visited the hospital again with the same symptoms but with extremely lower serum sodium concentration (106 mmol/L). Due to the extremely low serum sodium concentration the proband was presented with frequent convulsion. Pathological test showed that there is no abnormality in cerebral spinal fluid (CSF) as well as in the function of the endocrine gland. Proband also presented with chronic renal failure (creatinine concentration:155 μmol/l, blood urea nitrogen concentration: 14.7 mmol/l). Blood test identified that the proband had mild anemia (hemoglobin:80 g/L), which is frequently observed in Chinese patients (Liu et al., 2011). Proband’s biochemical test showed high level erythrocyte protoporphyrin, urine PBG, and uroporphyrin, which confirmed the clinical diagnosis of AIP. We recommended and treated the proband with administration of 250 gm of glucose given as intravenous injection as well as with restricted taking of fluid the proband get better and gained consciousness. After treatment the proband’s serum sodium concentration level was normal (137 mmol/L).

In this family, we identified only one patient (Proband) and proband’s father and mother is normal. Hence, in order to understand the molecular genetic basis of the disease in this family we undertaken a whole exome sequencing and confirmatory Sanger sequencing.

## Materials and Methods

### Whole Exome Sequencing

Genomic DNA was extracted from peripheral blood using a QIAamp DNA Blood Mini Kit (Qiagen, Hilden, Germany) according to the manufacturer’s instructions. All three family members (parents, proband) were subjected to exome sequencing. Sequences were captured by Agilent SureSelect version 4 (Agilent Technologies, Santa Clara, CA, United States) according to the manufacturer’s protocols. The enriched library was sequenced on an Illumina HighSeq2000. The sequencing reads were aligned to GRCh37.p10 using Burrows-Wheeler Aligner software (version 0.59). We then performed local realignment and base quality recalibration of the Burrows–Wheeler aligned reads using the GATK IndelRealigner and the GATK Base Recalibrator, respectively^2^. Single-nucleotide variants (SNV) and small insertions or deletions (indel) were identified by the GATK Unified Genotyper (see footnote 2). Variants were annotated using the Consensus Coding Sequences Database (20130630) at the National Center for Biotechnology Information.

### Method of Mapping, Genotype, SNP Calling and Indel Calling

Image analysis and base calling were performed using the Illumina Pipeline. Indexed primers were used for the data fidelity surveillance. SOAP aligner (soap2.21) was used to align clean reads to the human reference genome (hg19) with maximum 3 mismatches, the parameters were set as ‘-a -b -D -o -u -2 -t -v 3 -l 35 -s 40 -m 0 -x 500 -p 4 -r 1 -n 0’. Based on the results from SOAPaligner, software SOAPsnp (v1.05) was used to assemble the consensus sequence and call genotypes in target regions. The following parameters were set: -Q k -i -d -o -r 0.0005 -e 0.001 -u -L 90 -T -s -2,where ‘-T’ used the targeted and flanking regions. We filter candidate SNPs with the following criterion: snp quality ≧20, sequencing depth ≧4-fold, estimated copy number ≦2 and the distance between two SNPs is larger than 5 insertions and deletions (indels) in the exome regions were identified through the sequencing reads. We aligned the reads to the reference genome by BWA (0.6.2), the parameters were set as ‘-I -L -l 31 -i 15 -k 2 -t 6 -e 63’, and passed the alignment result to GATK, to identify the breakpoints, the parameters were set as ‘mismatch Fraction = 0.05, lod = 5, maxReadsF or Realignment = 30000, maxReadsInRam = 1000000′.

**FIGURE 1:**
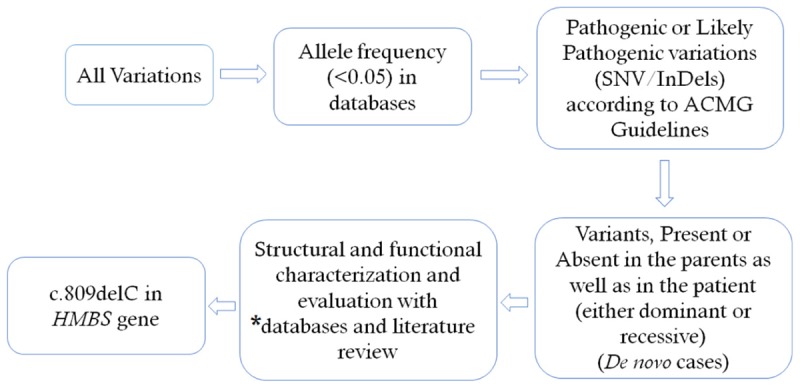
Filtering process for pathogenic mutations in all variations obtained by exome sequencing. ^∗^Databases used: dbSNP, Hapmap, 1000 Genomes Project and University’s in–house database of ∼500 Chinese people. SNV, single nucleotide variation: Indel, small insertion and deletion.

We selected variations obtained from exome sequencing with minor allele frequencies <0.05 in any of the following databases (dbSNP, Hapmap, 1000 Genomes Project) and our in-house database for ∼30,000 Chinese Han samples. We also selected pathogenic and likely pathogenic variations according to the ACMG guidelines (Richards et al., 2015). We further compared the remaining deleterious variations in the patient with variations carried by her unaffected parents and the gene’s functions with the references of OMIM and literature. For the detailed filtering process, please see **Figure 1**. Exome sequencing matrices are detailed given in Supplementary Table S1. Please see Supplementary Table S2 for the final list of heterozygous variants found in whole exome sequencing.

### Sanger Sequencing

To validate putative mutations, Sanger sequencing was performed. Primers flanking the candidate loci were designed based on the reference genomic sequences of the Human Genome from GenBank in NCBI and synthesized by Invitrogen, Shanghai, China. PCR amplification was carried out in an ABI 9700 Thermal Cycler. PCR products were directly sequenced on an ABI PRISM 3730 automated sequencer (Applied Biosystems, Foster City, CA, United States). Sequence data comparisons and analysis were performed by DNASTAR SeqMan (DNASTAR, Madison, WI, United States).

The heterozygous novel mutations identified through whole exome sequencing was verified through Sanger sequencing using the following primers: F1 5′-ACCCCATGACGCTTAATCAGGC-3′, R1 5′-GCAGCTTCTCACCCGGTTCTG-3′. The reference sequence NM_000190 of *HMBS* was used.

### Identification of Novel Mutation in *HMBS* Gene

**FIGURE 2:**
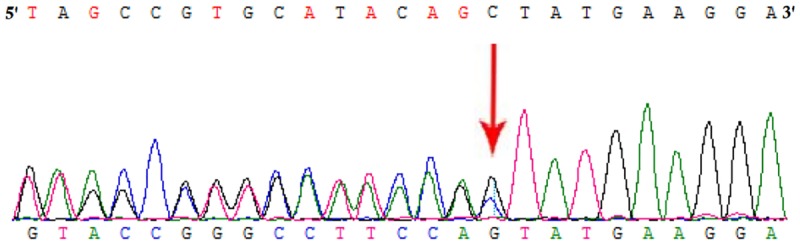
Partial DNA sequences in the *HMBS* by Sanger sequencing of the proband [NM_000190]. Arrows point to the mutations. The proband has c.809delC mutation.

Whole exome sequencing and Sanger sequencing identified a heterozygous novel single nucleotide deletion (c.809delC) in exon 12 of *HMBS* gene (**Figure 2**). This mutation was results into frameshift followed by formation of a truncated (p.Ala270Valfs^∗^2) HMBS protein product by formation of a premature stop codon. These findings lead us to suggest that the novel mutation found in the proband in this Chinese family may be the cause of the disease. This mutation was not present neither in the Exome Variant Server of the NHLBI-ESP database or in the 1000 Genomes database. It was also not detected in 100 normal Chinese control individuals.

### Structure Analysis

**FIGURE 3:**
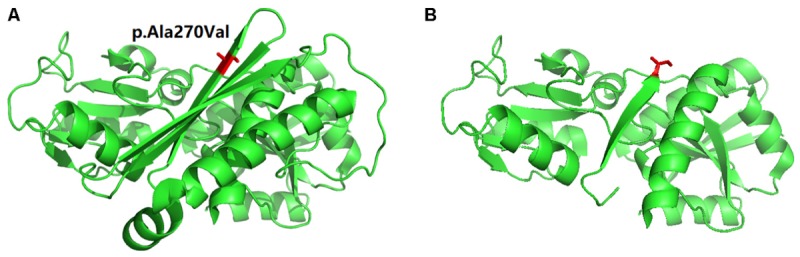
Schematic presentation of Protein 3D model. **(A)** The location (in “Red” color) of the mutation in the wild type protein. **(B)** The structure of the mutant protein upon frameshift mutation.

In order to understand the effect of this novel mutation c.809delC; p.Ala270Valfs^∗^2 on HMBS protein structure, we used the PDB ID: 3ECR. The location of the mutation in the wild type protein and the structure of the mutant protein upon frameshift mutation has schematically presented in **Figures 3A,B**.

## Discussion

Acute intermittent porphyria is a rare autosomal dominant form of acute porphyria. Germline mutations in *HMBS* gene causes AIP with partial deficiency of hydroxymethylbilane synthase PBGD. However, AIP also may be life-threatening because of the presence of high level of porphyrin precursors in the nervous systems (Hrdinka et al., 2006). Clinically AIP is extremely heterogenous with a spectrum of disease phenotype which leads to misdiagnosis at the earlier stage.

Till now, approximately 360 *HMBS* gene mutations has been reported to be associated with AIP^3^. In this study, we identified a novel *HMBS* gene mutation (c.809delC; p.Ala270Valfs^∗^2) in a Chinese family. In China, due to poor awareness, proper and early clinical diagnosis for patients with AIP is very challenging. Moreover, generally, urinary δ-aminolevulinic acid (ALA) level and plasma emission peak were playing the key role for clinical diagnosis of AIP (Balwani and Desnick, 2012; Kong et al., 2013). But, in China, due to lack of these tests, genetic screening is obvious. Whole exome sequencing is the best possible way to understand the molecular genetics causes of the disease in patients with AIP.

Here, whole exome sequencing identified a novel heterozygous single nucleotide deletion c.809delC which leads to frameshift followed by formation of a truncated (p.Ala270Valfs^∗^2) HMBS protein product by the formation of a premature stop codon. This report is the first family-based genetic study of Chinese AIP patients with deletion in *HMBS* gene in Mainland China. In this family, we identified only one patient (Proband) and proband’s father and mother is normal, establishing that the mutation is a *de novo* mutation.

In addition, here, the identified candidate mutation (c.809delC) causes formation of a truncated HMBS protein (p.Ala270Valfs^∗^2) of 272 amino acids instead of the wild type HMBS protein which consists of 361 amino acids. So, this truncated HMBS protein lacks of 89 amino acid compared to the wild type HMBS protein. Therefore, this truncated HMBS protein will be unstable and causes haplo-insufficiency which finally results into AIP. This is the molecular consequences of this mutation. Hence, this is a *“loss-of-function”* mutation. According to the variant interpretation guidelines of the American College of Medical Genetics and Genomics (ACMG), this variant is classified as *“likely pathogenic”* variant (Richards et al., 2015).

Due to extreme phenotypic heterogeneity of AIP patients with *HMBS* mutations, we are also discussing here the genotype-phenotype correlation. Although, *HMBS* associated AIP cases are very common in western population, but in Chinese population it is very rare. In addition, in Chinese population, AIP associated with mild anemia is rarest. So, drawing the conclusion for the genotype-phenotype correlation is really challenging because phenotype is more population specific rather than mutation type or location. So, comparing between Chinese AIP patients with *HMBS* mutation and AIP patients of Western population are less useful for genotype-phenotype correlation study. In our future study, we are planning to work with a large cohort of Chinese AIP patients with *HMBS* mutations which would have a great sense or scientific significance for genotype-phenotype correlation study.

Germline mutation in *HMBS* gene leads to partial deficiency of HMBS protein followed by loss of the heme biosynthetic pathway which in turn causes accumulation of ALA and PBG. In AIP patients, consecutive loss of electrolyte balance could lead to brain damage by causing encephaloedema or seizures. Excessive accumulation of ALA and partial activity of heme leads to hypothalamic damage. In AIP patients, anemia, could be caused due to low rate of biosynthesis of heme in erythroid precursor cells from bone marrow (Dos Santos et al., 2013). Proper awareness and genetic screening leads to early recognition and clinical diagnosis of AIP and prompt treatment which is important for clinical management. In China, due to unavailability of heme treatment, diet with high carbohydrate and restricted fluid intake causes gradual recovery of the AIP patients. In addition, follow up studies with AIP patients also a key factor for proper clinical management. In addition, our present study established the application of high throughput exome sequencing technology for understanding the genetic cause of the disease in AIP patients.

## Concluding Remarks

Here, we report a clinical case of rare AIP in a Chinese family. This case highlights the importance of whole exome sequencing for proper clinical diagnosis of the AIP associated with germline mutation in *HMBS* gene. Hence, molecular genetic screening of *HMBS* gene should be considered to confirm the patients clinically diagnosed with AIP. Our present study also emphasizes the significance of the high quality genetic analysis by whole exome sequencing in the molecular diagnosis of rare inherited mendelian disorders with extreme phenotypic heterogeneity.

## Ethics Statement

We obtained written informed consent for genomic analysis of the patient and his family members in accordance with the Declaration of Helsinki. The project was approved by the ethics committee of the Third Affiliated Hospital of Sun Yat-sen University and informed consent was obtained from all participants. The proband and his family members provided written informed consent for the publication of the patient’s identifiable information.

## Dataset with Accession Number

Database: Genome Sequence Archive, BIG Data Center in Beijing Institute of Genomics (GEO). Accession number: CRA000851. URL: http://bigd.big.ac.cn/gsub/submit/gsa/list.

## Author Contributions

YZ, JX, and DL: patient workup. SB and SL: genetic analysis. SB, YZ, JX, and SL: drafting the manuscript. SB, YZ, JX, SL, and DL: final approval for the version to be published and agreement to be accountable for all aspects of the work.

## Conflict of Interest Statement

The authors declare that the research was conducted in the absence of any commercial or financial relationships that could be construed as a potential conflict of interest.

## Footnotes

1 www.hgmd.cf.ac.uk

2 broadinstitute.org

3 http://www.hgmd.cf.ac.uk/ac/index.php
