# Supplementary material for: Whole Exome Sequencing Identified a Novel Heterozygous Mutation in HMBS Gene in a Chinese Patient With Acute Intermittent Porphyria With Rare Type of Mild Anemia
Source: Front Genet. 2018 Apr 20;9:129. doi: 10.3389/fgene.2018.00129 (PMC5920022; doi:10.3389/fgene.2018.00129)
Supplement: Supplementary file 1 [file Table_1.DOCX]

**Supplementary Table S1: Whole exome sequencing metrics.**

| Original data output (Mb) | 10086.18 |
| --- | --- |
| Target area length (BP) | 58882239 |
| Target area coverage | 99.66% |
| Average depth of target area (X) | 102.25 |
| The proportion of the average depth of >10X in the target area is（X） | 96.95% |
| The proportion of the average depth of >20X in the target area is | 91.73% |
