# Supplementary material for: Whole Exome Sequencing Identified a Novel Heterozygous Mutation in HMBS Gene in a Chinese Patient With Acute Intermittent Porphyria With Rare Type of Mild Anemia
Source: Front Genet. 2018 Apr 20;9:129. doi: 10.3389/fgene.2018.00129 (PMC5920022; doi:10.3389/fgene.2018.00129)
Supplement: Supplementary file 2 [file Table_2.DOCX]

**Table S2: List of the variant(s) identified on Whole Exome Sequencing.**

| Number | Genes | RefSeq | Nucleic Acid Alteration | Amino Acid Alteration | Mutation  location | Zygosity * | Chr_location | **RS** | Frequency in | | Mutation Function |
| --- | --- | --- | --- | --- | --- | --- | --- | --- | --- | --- | --- |
|  |  |  |  |  |  |  |  |  | 1000-genome’s | Chinese Database |  |
| 1 | *CPOX* | NM_000097 | c.990A>G | p.Glu330Glu | EX5/CDS5 | Het | chr3:98304467 | rs1729995 | 0.3498 | 0.5188 | - |
| 2 | *CPOX* | NM_000097 | c.880G>A | p.Val294Ile | EX4/CDS4 | Het | chr3:98307630 | rs2228056 | 0.1484 | 0.4017 | - |
| 3 | *FECH* | NM_001012515 | c.939A>G | p.Pro313Pro | EX9/CDS9 | Hom | chr18:55221648 | rs536560 | 0.5641 | 0.5904 | - |
| 4 | *FECH* | NM_001012515 | c.816C>G | p.Pro272Pro | EX7/CDS7 | Hom | chr18:55226383 | rs536765 | 0.5476 | 0.5327 | - |
| 5 | *HMBS* | NM_000190 | c.606G>T | p.Val202Val | EX9/CDS9 | Het | chr11:118962230 | rs1131488 | 0.3031 | 0.4378 | - |
| 6 | *HMBS* | NM_000190 | c.809delC | p.Ala270ValfsX2 | EX12/CDS12 | Het | chr11:118963505 | - | 0 | 0 | Likely pathogenic |
| 7 | *PPOX* | NM_000309 | c.911G>A | p.Arg304His | EX9/CDS8 | Het | chr1:161139738 | rs36013429 | 0.0879 | 0.1103 | - |

Zygosity : Hom represents a homozygous mutation, Het represents a heterozygous mutation, Hemi represents a Hemizygous mutation.

Frequency in 1000 genome’s: Occurrence frequency in 1000 genomes project.

Chinese Database: Occurrence frequency in local database of university (> 500 Chinese).
